# Identifying Interactive Factors That May Increase Crash Risk between Young Drivers and Trucks: A Narrative Review

**DOI:** 10.3390/ijerph18126506

**Published:** 2021-06-16

**Authors:** Melissa R. Freire, Cassandra Gauld, Angus McKerral, Kristen Pammer

**Affiliations:** The School of Psychology, Faculty of Science, The University of Newcastle, Callaghan, NSW 2308, Australia; Cass.Gauld@newcastle.edu.au (C.G.); Angus.McKerral@newcastle.edu.au (A.M.); Kristen.Pammer@newcastle.edu.au (K.P.)

**Keywords:** truck safety, heavy vehicle, young driver, risky driving behaviour, crash risk, driver education

## Abstract

Sharing the road with trucks is associated with increased risk of serious injury and death for passenger vehicle drivers. However, the onus for minimising risk lies not just with truck drivers; other drivers must understand the unique performance limitations of trucks associated with stopping distances, blind spots, and turning manoeuverability, so they can suitably act and react around trucks. Given the paucity of research aimed at understanding the specific crash risk vulnerability of young drivers around trucks, the authors employ a narrative review methodology that brings together evidence from both truck and young driver road safety research domains, as well as data regarding known crash risks for each driving cohort, to gain a comprehensive understanding of what young drivers are likely to know about heavy vehicle performance limitations, where there may be gaps in their understanding, and how this could potentially increase crash risk. We then review literature regarding the human factors affecting young drivers to understand how perceptual immaturity and engagement in risky driving behaviours are likely to compound risk regarding both the frequency and severity of collision between trucks and young drivers. Finally, we review current targeted educational initiatives and suggest that simply raising awareness of truck limitations is insufficient. We propose that further research is needed to ensure initiatives aimed at increasing young driver awareness of trucks and truck safety are evidence-based, undergo rigorous evaluation, and are delivered in a way that aims to (i) increase young driver risk perception skills, and (ii) reduce risky driving behaviour around trucks.

## 1. Introduction

Sharing the road with trucks and heavy vehicles is associated with greater risk of serious injury or death for drivers of passenger vehicles. In Australia in 2019, 188 people died from 173 crashes involving heavy trucks; and this figure represents a 27.2% increase in fatalities compared to 2018 [1]. Collisions involving trucks are 2.6 times more likely to result in a fatality [2], and passenger vehicle occupants are 10 times more likely than truck occupants to suffer serious or fatal injuries [3]. Crash statistics indicate that 78% of fatalities and 76% of injuries resulting from passenger vehicle-truck collisions are sustained by occupants of the passenger vehicle [4].

An in-depth review of fatal truck-passenger vehicle collisions in the United States of America (U.S.) reveals that 67% of fatal crashes are attributable to the unsafe driving behaviours of passenger vehicle drivers, with the most prevalent behaviours being: veering out of their lane (19.9%); failing to give way (14.4%); speeding (14.1%); and driver inattentiveness (8.7%) [5]. Other research suggests these figures may indeed be even higher, stating that passenger vehicle contribution to collisions with heavy vehicles is more likely to be around 80–83% [6,7].

Worldwide, road trauma is the leading cause of death among young people aged under 29 years [8]. In Australia, young drivers are grossly over-represented in road crash statistics. In 2020, of the 1106 people killed on Australian roads, 208 were young people aged 17 to 25 years (approx. 19%), despite this age demographic constituting only approximately 12% of the population [9]. The highest crash risk period for a young person is immediately after obtaining a provisional driver licence (where the driver is unsupervised) and increased risk continues until around age 24 years [10]. Generally speaking, young male drivers tend to engage in more risky driving behaviours than young female drivers [11], and, as such, young males under 25 years, worldwide, are almost three times more likely to be killed in a road traffic crash than young females [8].

Given the over-representation of young drivers (under 25 years) in crash statistics [8,9] and the increasing number of trucks on the road [12], there is a need to understand the possible contributing factors for crash risk between young drivers and trucks so as to reduce serious injury and fatality risk for these driver populations. While it is acknowledged that young drivers are seemingly unaware of the limitations of heavy vehicles, or how their own driving behaviour may contribute to increased crash risk around trucks [13], a thorough literature search reveals that there is little empirical research into whether a cumulative crash risk exists for young drivers aged 17–25, when driving in the vicinity of trucks or heavy vehicles.

The overall purpose of this review is to bring together evidence regarding the known hazards and limitations of trucks that increase likelihood of collision, and evidence regarding human factors that influence young driver road safety and are known to increase likelihood of collision. A paucity of available research investigating this interactive relationship between truck collision risk and young driver collision risk makes it difficult to undertake a full systematic review, so a narrative review was undertaken to weave together a narrative based on evidence from within these different fields of research. A narrative review approach is recommended when a breadth of analysis across multiple areas is required, as it is deemed more effective than a systematic review when research topics require a wider scope than what can be achieved within the rigid confines of a systematic review [14].

This narrative review aims to determine what known risk factors are likely to increase collision risk between trucks and young drivers by (i) providing an overview of the evidenced limitations of trucks, as well as the key collision risk factors identified by truck drivers and road safety experts, and (ii) conducting a review of the known human factors affecting young driver behaviour and discussing how such factors may increase collision risk between young drivers and trucks. We then provide recommendations for how interacting factors may then be targeted in young driver education initiatives that are designed to minimise young driver collision risk with trucks.

## 2. Narrative Review Methodology

Given that this review investigated a breadth of literature across multiple research domains, an iterative search process was used whereby a number of thematic searches were conducted. Inclusion and exclusion criteria for particular search terms was determined by outcomes of previous searches. Given that we were accessing academic, industry-based, and government literature, all searches were conducted using a large Australian University database, as well as Google and Google Scholar. A search in the reference lists of the articles identified in these searches provided some additional literature that was also included in the current narrative review.

Our initial search, using the University database, sought to identify existing literature regarding the known interactive crash risk between trucks and young drivers. Key search terms included ‘young driver’, ‘novice driver’, ‘truck’, ‘heavy vehicle’, ‘crash risk’ ‘crash statistics’, ‘crash report’, ‘road safety’. An extensive review of search results revealed that there were no peer-reviewed, published journal articles that specifically addressed the known crash risk between trucks and young drivers specifically, however search results did provide information about crash risk for trucks and crash risk for young drivers separately. Google Scholar and Google searches using the same terms produced some media articles that suggested a specific crash risk between trucks and young drivers, however these articles were not peer reviewed or published in academic journals. Therefore, subsequent searches, using a University database and Google/Google Scholar, were conducted regarding performance limitations of trucks and the relationship with road safety, using the following search terms: ‘truck’, ‘heavy vehicle’, ‘limitation’, ‘performance limitation’, ‘crash risk’, ‘crash report’, ‘road safety’. As well as providing information regarding performance limitations of trucks to inform Section 3 of this paper, this search also revealed literature regarding the road safety and crash risk perceptions of truck drivers and industry experts, which informed Section 4.

Outcomes from the initial search, coupled with our academic understanding of young driver crash risk, were then the basis for an additional search that would inform the weaving of a narrative around the likely crash risk of young drivers around trucks. Using the University database as well as Google/Google Scholar, we entered the following search terms: ‘young driver’, ‘novice driver’, ‘inexperienced driver’, ‘driver age’, ‘human factor’, ‘driving’, ‘driving behaviour’, ‘risky driving’, ‘driver inattentiveness’, ‘situation awareness’, ‘hazard perception, ‘neural function’, ‘neural maturation’, ‘executive function’. This informed Section 5.

As we were also interested in potential avenues for young driver education around trucks and truck safety, we wanted to know what current education initiatives existed in Australia. Therefore, we conducted a final search using a University database and Google/Google Scholar, including the terms: ‘young driver’, ‘novice driver’, ‘learner driver’, driver licensing’, ‘driver education’, ‘Australia’, ‘truck safety’, ‘road safety’, ‘Australian Trucking Association’. This informed Section 6.

## 3. Performance Limitations of Trucks and Associated Road Safety Risks

Safe driving behaviour around trucks requires that passenger vehicle drivers are aware of the physical and operational limitations of heavy vehicles due to their size, mass and weight, and are able to engage in necessary adaptive driving techniques to accommodate such limitations [15]. Here we outline the key performance limitations of trucks with regards to driver visibility, braking and stopping distance, and turning, and consider what necessary accommodations can be made by other road users to minimise crash risk.

Truck driver visibility is impeded by the size of the vehicle. Trucks have greater blind spot areas than passenger vehicles, and impaired visibility in these areas increases crash risk [16]. A blind spot area is defined as an area around the perimeter of the vehicle that cannot be seen by the driver using standard windows or mirrors [17]. Truck drivers have four main blind spot areas: immediately in front of the truck; behind the door on the driver side of the vehicle, the length of the passenger side of the vehicle, and directly behind the truck. To minimise crash risk associated with lane changing or merging, other motorists need to avoid driving in these blind spot areas [16] and need to be mindful of the intentions of truck drivers if they are situated in a truck blind spot. Although there is little research into motorists’ knowledge of truck and heavy vehicle blind spots, a study conducted in Malaysia found that when questioned on their knowledge of the location of truck blind spots, less than half of the 100 surveyed respondents were aware of blind spot locations to the front, left and right perimeters of a heavy vehicle [16]. Furthermore, when asked to recall whether they remembered learning about heavy vehicle blind spots from driver schools when learning to drive, 69% of respondents recalled receiving little or no driver education regarding blind spots of heavy vehicle drivers [16]. When we consider that road user manuals in Australia provide only a cursory overview of truck blind spots (e.g., the 212 page New South Wales (NSW) Road User Handbook [18] has half a page dedicated to truck and bus blind spots), young drivers in Australia are likely to similarly have little awareness of the limited truck driver visibility due to blind spots. As such, targeted education may be necessary to increase awareness and understanding to minimise crash risk associated with passenger vehicles driving in truck blind spots.

Truck stopping distance is directly affected by the mass of the vehicle, and this is compounded by the weight of the vehicle’s load. For example, when travelling at 60 km per hour, the average necessary stopping distance for a truck is 83 m, compared to 73 m for most other passenger vehicles. The difference in stopping distance between trucks and passenger vehicles increases with increased vehicle speed. For example, when travelling at 100 km per hour, trucks require an average safe stopping distance of 185 m, which is 28 m more than the required stopping distance of a passenger vehicle at the same speed [17]. Necessary considerations for other drivers with regards to truck stopping distance are to ensure they do not travel too closely behind trucks, they leave enough room behind them when overtaking or merging, and they avoid braking heavily when driving in front of trucks [17]. External factors such as wet weather will increase truck stopping distances, so greater care needs to be taken in such conditions to minimise crash risk.

A large-scale U.S. analysis of recorded dash-cam footage from 100 participatory passenger vehicles was conducted to identify what specific heavy vehicle and passenger vehicle driver behaviours contributed to increased collision risk [19]. This analysis identified 246 critical incidents between heavy vehicles and passenger vehicles, where critical incidents were defined as “unexpected events resulting in a close call or requiring fast action (evasive maneuver) on the part of a driver to avoid a crash” [19] (p. ii). The passenger vehicle driver was deemed to be at fault in 64% of these critical incidents. Of these passenger vehicle at-fault incidents, the most frequently identified incident types were late braking for stopped/stopping traffic (41.3%) and lane change without sufficient gap (21.7%). A majority of passenger vehicle at-fault incidents were attributed to the passenger vehicle not leaving sufficient headway between vehicles to accommodate the longer stopping distance of the heavy vehicle [19]. This suggests that a majority of passenger vehicle at-fault incidents related to an unawareness of or failure to consider truck performance limitations associated with braking and stopping.

The mass of trucks also affects manoeuvrability, and this is particularly relevant when trucks are turning. Limited manoeuvrability means that trucks need more space when turning, and other drivers need to give way (yield) to ensure the truck can turn safely. Hanowski, et al. [19] found that 14.4% of potential heavy vehicle–passenger vehicle collisions were associated with truck turning manoeuvrability. Trucks are legally allowed to use more than one lane to complete their turn and drivers need to ensure that they do not try to cut into the lane beside the turning truck, as this can prevent the truck driver’s ability to safely complete the turn [17]. While trucks are fitted with a sign reminding motorists to give way to trucks while turning, there is little education provided regarding this requirement; although some road users’ handbooks do explicitly show an image of the “Do not overtake turning vehicles” sign, others do not, and most manuals do not provide sufficient detail regarding the reasons why it is essential to give trucks space to turn safely or the consequences to the passenger vehicle occupants if they are too close to a turning truck or heavy vehicle (for example, see [18]).

The size and manoeuvrability limitations of heavy vehicles provide a challenge for other road users, and evidence suggests that these factors have directly contributed to increased road safety collision risks between truck and passenger vehicle drivers. While a great deal of research has been conducted nationally and internationally to understand truck safety from the context of the state of the truck driver (e.g., fatigue, age, physical and mental health, drug use, and experience; [20,21,22]), environmental factors (e.g., time of day; [23,24]), the impact of infrastructure (e.g., road design, truck design and vehicle checking, and speed limits; [25,26]), as well as regulatory and management issues (e.g., shift length, payment, management style and expectations; [21,27]), little research has investigated other road users’ knowledge and perceptions of trucks or how the driving behaviour of passenger vehicle drivers directly contributes to increased collision risk with trucks. Research that has been conducted in this regard is specific to the U.S. and Canada, and is rather dated (e.g., [5,28]), thus does not take into account recent initiatives aimed at decreasing young driver crash risk, such as graduated driver licensing programs [29] and demonstrated strategies to provide targeted road safety messaging (e.g., [30]). To reduce crash risk between trucks and passenger vehicles, it is necessary to understand the contributory risk of passenger vehicles, particularly with regards to passenger vehicle driver awareness and behaviour and intention around trucks, which can be leveraged to deliver targeted education initiatives that will ultimately reduce crash risk.

## 4. Perceptions of Road Safety around Trucks

An in-depth qualitative study was conducted in the U.S. to understand what experienced truck drivers and collision analysts determined were the key risk factors for crashes involving trucks and other heavy vehicles. This study found that the main perceived crash risk was unsafe driving behaviours of other motorists around trucks, which collision experts and experienced drivers agreed primarily stemmed from passenger vehicle drivers being ignorant to the performance limitations of trucks and heavy vehicles [5]. Experts stated that most passenger vehicle drivers were unaware of the limitations of large trucks, particularly with regards to truck visibility, braking, and acceleration, and they lacked knowledge of the relationship between mass and velocity that is associated with stopping distance. Furthermore, experienced truck drivers suggested that the most common unsafe driving behaviours of other motorists that contributed to increased collision risk included (in order of most cited factors): (i) driving in truck blind spots; (ii) making abrupt lane changes and improperly merging in front of a truck; and (iii) driving inattentively [5].

In contrast to expert perceptions of truck crash risk, passenger-vehicle drivers’ perceptions of increased risk around trucks and general truck safety focused more on the dangers of trucks and the potentially dangerous actions of truck drivers. A large-scale Canadian survey of 1668 motorists revealed that the key factors motorists perceived to contribute to increased collision risk around trucks (in order of mean concern rates) were: material thrown up by truck tires, trucks swaying, truck stopping distance, difficulty seeing around trucks, cargo falling off, truck driver error, wind turbulence caused by trucks, time required to pass a truck, and truck mechanical failure [28]. This finding suggests that while motorists do generally appear to have safety concerns when driving around trucks, their concerns are more focused on the dangers directly caused by trucks and the behaviour of truck drivers, but are generally ignorant to the potential dangers of passenger vehicles when driving in the vicinity of trucks. Consequently, when driving around trucks, passenger vehicle drivers are more likely to be focused on what they perceive to be dangers specifically associated with heavy vehicles, rather than focusing on the contributory crash risk factors stemming from their own driving behaviours.

A more recent survey of U.S. motorists investigated the relationships between motorists’ perceptions of trucks and truck safety and their own driving experiences and behaviours [31]. This study demonstrated that motorists’ own driving behaviours influenced their perceptions of trucks and truck safety, such that drivers who engaged in more risky driving behaviours themselves, and who had a higher propensity for sensation seeking and risk taking, were more likely to have an overall less negative perception of trucks [31,32]. This is an interesting finding, as it considers the interaction between human factors associated with driving and motorists’ perceptions of trucks and truck safety.

Further research is needed to determine how such perceptions affect the actual driving behaviour of these risky drivers around trucks. For example, these drivers may have an inflated impression of truck driver ability to react in dangerous situations. This may result in unfounded assumptions that they do not need to modify their own risky driving behaviours around trucks to accommodate truck limitations; this could potentially increase the risk of a truck-passenger-vehicle collision. This may provide an avenue to target driver beliefs and intentions associated with risky driving behaviours around trucks via driver education or persuasive messaging, areas for future research that we will discuss in the next section.

## 5. Human Factors Underlying Young Driver Behaviour

A young driver’s inability to perceive and comprehend the limitations of trucks could be attributed to limitations in situation awareness and hazard perception that are associated with driver inexperience and incomplete neurocognitive maturity, however a thorough review of the literature reveals that this interaction has never been empirically explored. As such, we also discuss situation awareness and hazard perception limitations of young drivers, and how this relates to reduced awareness of truck limitations and make suggestions for future research in this area. Road safety research conducted from a psychological perspective identifies human factors as key predictors of increased motor vehicle crash risk, contributing to approximately 95% of road traffic crashes [33]. With regards to driving behaviour, human factors can be classified as (i) enduring factors that affect driving ability (e.g., aging, drug abuse); (ii) immediate factors that reduce driving capability (e.g., fatigue, distraction); (iii) enduring factors that promote risk-taking (e.g., optimism bias, habitual speeding); and iv) immediate factors that promote risk-taking (psychotropic drugs, compulsive acts) [33]. With regards to collision risk, the contribution of human factors can be further delineated into proximal and distal factors. Proximal factors are directly associated with collision likelihood, and are predominantly associated with driver intent and aberrant driving behaviour, such as speeding, driving aggressively and driving under the influence of alcohol or drugs [32]. In this context, speeding and traffic violations are evidenced to be stable predictors of collision likelihood [32]. Distal human factors more indirectly predict collision likelihood, and include psychosocial factors associated with risky driving behaviour, such as sensation seeking, aggression, individual personality characteristics, and optimism bias [32]. In addition, impaired or underdeveloped situation awareness and hazard perception are associated with increased crash risk [34,35].

While all these aforementioned factors are associated with increased crash risk for the general driving population, they are likely to be especially predictive of increased crash risk for vulnerable sub-populations of drivers such as young drivers, and particularly young male drivers [36,37]. Overarching factors associated with increased crash risk for young drivers include: (i) they have not yet reached full neurocognitive maturity, which is associated with reduced executive function and cognitive capacity to react in scenarios that impose a high cognitive workload, such as driving [38,39]; (ii) they have a lack of driving experience, which is associated with underdeveloped situation awareness and hazard perception skills [34,35]; and, (iii) they are more likely to engage in both intentional and unintentional risky driving behaviours [36].

To reduce crash risk between trucks and young drivers, each of these three overarching factors needs to be appropriately understood and addressed through targeted, evidence-based educational initiatives. Successful approaches to addressing neurocognitive maturity and driver inexperience include delaying the ability to obtain a driver licence, thus ensuring that drivers have ample opportunity to gain driving experience in a number of driving situations while still under the instruction or guidance of a fully-licensed driver. These objectives have largely been addressed by introducing graduated driver licensing schemes in all states and territories of Australia, which has resulted in a significant reduction in young driver fatalities [36,40]. However, risky driving behaviours also need to be understood within a human factors framework to enable promotion of safe driver behaviour via contextually relevant driver education and appropriately targeted road safety messaging.

To gain a holistic understanding of the factors that could put younger drivers at greater risk of collision with trucks and other large vehicles, it is necessary to understand how known risky driving behaviours that are most associated with collision risk for young drivers might especially exacerbate their risk of collision with trucks. These known risky driving behaviours include the presence of peer passengers, speeding, night driving, and driver distraction, particularly due to smartphone use. Issues associated with young driver inexperience, neurocognitive immaturity, and risky driving behaviours will now be discussed and considered in terms of increased collision risk around trucks.

### 5.1. Young Driver Inexperience: Underdeveloped Situation Awareness and Hazard Perception

Safe driving behaviour around trucks requires an awareness of the physical and operational limitations of heavy vehicles due to their size, mass and weight, as well as an awareness of the adaptive driving techniques of passenger vehicle drivers around trucks to minimise collision risk [5,13]. An understanding of the limitations of trucks and other heavy vehicles generally builds as motorists gain driving experience and consequently develop skills associated with situation awareness and hazard perception. Due to their limited experience, young drivers are not actively aware of the limitations of heavy vehicles, nor are they aware of how their own driving behaviour may contribute to increased crash risk around trucks [13]. We propose that a young driver’s inability to perceive, comprehend, and adjust their own driving behaviours to accommodate the limitations of trucks can be partially attributed to limited situation awareness and hazard perception that is associated with driver inexperience.

#### 5.1.1. Situation Awareness

The characteristics identified as being associated with truck crashes, such as failure to give way to turning trucks, overtaking or merging in the vicinity of trucks without leaving ample stopping distance, driving in truck blind spots, and driver inattentiveness, implicate deficiencies in situation awareness by passenger vehicle drivers, a deficiency that is sustained due to relatively few trucks on the road. Here then it is conceivable that some of the road safety violations around trucks occur because of a lack of situation awareness around trucks, and little opportunity to organically develop necessary awareness due to infrequent encounters with trucks when driving.

Situation awareness can be conceptualised as the perception of relevant features within the environment, comprehension of their meaning, and projection of their state into the near future [41]. Safe driving behaviour is dependent upon awareness at these each of these levels of situation awareness [42]. Improved situation awareness is dependent upon driver experience; as drivers become more experienced many of the functions associated with perception and comprehension of features within the driving environment become automated, thus allowing the driver to devote more attention to projecting the state of environmental features into the near future [43]. Due to limited driving experience, young drivers typically demonstrate more effortful perception and comprehension of routine features associated with driving [44], and thus have limited attentional resources available for higher order situation awareness associated with recognition and anticipation of potential crash risks in the driving environment. Conversely, experienced and expert drivers demonstrate a high degree of automaticity of various features of driving, such as changing gears, scanning mirrors, checking blind spots when changing lanes, thus leaving greater attentional resources available to project dynamic changes in the driving environment and react appropriately to avoid collision [35,43]. This relative situation awareness of young, inexperienced drivers (under 25 years) and more experienced drivers (aged 25 years and older) has implications for the way each cohort responds to trucks on the road.

Given that early cognitive components of young drivers’ situation awareness that might facilitate perception of a truck in the vicinity are less likely to be automatic than for an older more experienced driver [43], young drivers are unlikely to have the necessary attentional resources available to adequately maintain higher order situation awareness and project the future state of features of the driving environment. When we consider that the impetus is placed on the passenger vehicle driver to take extra precautions when driving in the vicinity of a heavy vehicle, a reduced situation awareness for young drivers has implications for how their own driving behaviour might directly contribute to an increased crash risk around trucks. They are less likely to recognise, anticipate and make necessary driving accommodations around trucks to minimise crash risks, for example, they may not recognise the dangers associated with driving in a truck’s blind spot and therefore not make the necessary accommodations to avoid doing so. As we discuss in the next section, this corresponds with what is understood about the influence of neurocognitive development on driving ability, whereby immature neurocognitive maturation is associated with difficulty in engaging in tasks that require high mental workload [38,39].

While there is evidence of young drivers having limited situation awareness [34], the relationship between the situation awareness of young drivers with regards to trucks and truck limitations has not yet been investigated, and the assumptions made here need to be informed by explicit evaluations of young driver behaviour around trucks. Future research is needed to assess the influence of young driver situation awareness on crash risk with trucks, as well as the explicit relationship between situation awareness and neurocognitive development. We recommend that investigation of the likely benefit in educating young drivers about truck limitations to improve situation awareness should be explored.

#### 5.1.2. Hazard Perception

Hazard perception is defined as the process of identifying hazardous features of the environment and assessing their threat [45] and can be understood as a component of a driver’s higher order situation awareness that similarly improves with driving experience [35,45,46]. Graduated licensing schemes throughout Australia acknowledge the importance of hazard perception as a necessary skill associated with safe driving practice, as evidenced by the requirement for learner and provisional drivers to complete hazard detection tasks prior to progressing to either a provisional or full licence [47]. The importance of hazard perception skills for safe driving practice is demonstrated by research indicating that failure to pass the hazard perception test is associated with a twofold increased crash risk [48].

In relation to safe driving practice around trucks, we can consider hazard perception as the ability to detect potential hazards associated with driving in the vicinity of trucks, in part due to the operational limitations of heavy vehicles. For example, when overtaking a truck, young drivers may not consider the longer stopping distance of a heavy vehicle. Consequently, the naïve younger driver may leave what they believe is a safe and appropriate distance for a passenger vehicle when merging back into the forward lane of the truck, but that distance would not allow the truck to stop safely in the event of an emergency. In this example, the young driver is not aware that their own driving behaviour may be hazardous for the truck driver, in that it increases the risk of collision in the event that both vehicles needed to brake suddenly. The young driver’s inability to detect this potential hazard can be explained within a framework of driver schemas. Schemas (established task-specific models) are developed as experience conducting a certain task increases [49]. In this case, the driver of a passenger vehicle will possess a schema of the driving task, which includes an understanding of the way other road-users behave [50]. The more sophisticated the schema, the faster and more accurately a driver will be able to respond to a predictable event. Both experiential (learned through on-road encounters) and explicit (driver training courses) knowledge regarding truck performance limitations and associated behaviour can improve a driver’s schema, which improves the quality of future similar interactions within the road environment [51,52].

Explicit knowledge training has been shown to improve both situation awareness [53] and hazard perception [45]. While there is no available evidence on the benefits of such training on improving a driver’s schema specifically for trucks and hazardous scenarios involving trucks, explicit knowledge training offers promise in improving situation awareness and hazard perception with regards to safe driving practice around trucks for young drivers. This provides young novice drivers–who have the least attentional resources available and the least sophisticated schemas for trucks–the opportunity to build and develop these resources in a safe simulated driving environment. Further research is needed to assess whether driver knowledge training around trucks and truck performance limitations can boost situation awareness and hazard perception skills, to assess whether such training can ultimately reduce crash risk for young drivers around trucks.

### 5.2. Young Driver Maturational Limitations in Neurocognitive Function

As we have identified, young drivers (under 25 years) have an increased crash risk compared to older drivers (over 25 years). This is evident in an analysis of novice drivers (who have held their driver licence for approximately two years, but may vary in age), where crash risk was found to be approximately twice as high for younger (16–19 years) versus older novice drivers (aged 20+ years) in their first few months of driving [54]. While increased experience is a recognised factor associated with decreased crash risk, consideration of experience alone does not sufficiently explain this difference in crash risk between younger and older novice drivers. Interestingly, research suggests that the age at which crash risk reduces is about 25 years [55,56], which is also the age at which the prefrontal cortex of the brain fully matures [57]. This points to another important consideration: the immaturity of young drivers’ neural function and how this influences driving ability, particularly in situations that are dependent upon increased mental workload. In a driving context, mental workload can be defined as a measure of neural resource capability required to satisfy driving task demands [58].

Neuroscientific studies have shown that the prefrontal cortex region of the brain is activated during driving tasks, for example transcranial direct current stimulation (tDCS) of the dorsolateral region of the prefrontal cortex can lead to safer driving behaviour [59]. The prefrontal cortex is activated for many executive functions associated with increased mental workload while driving, such as working memory, inhibitory control, set-shifting, judgment and decision making [38,39,60,61]. Functional neuroimaging studies have demonstrated the relationship between immature maturation of the prefrontal cortex and impaired driving ability under high mental workload. For example, using functional near infrared spectroscopy (fNIRS) to measure prefrontal cortex activity, Foy, et al. [38] demonstrated that during high mental workload tasks such as overtaking (a known high risk driving activity for young drivers; [36]), older drivers show greater prefrontal cortex activation than younger drivers. The authors suggest that this difference may be associated with a lack of maturation of this region for younger drivers, contributing to a reduced threshold for driving errors. Interestingly, the authors found that despite measurable differences in prefrontal cortex activation between older and younger drivers, there were no age differences in subjective reports of mental workload, such that younger drivers “do not feel that overtaking requires a degree of workload beyond their capabilities” [36] (p. 12). Differences in young drivers’ actual versus perceived mental workload capacity suggests that these drivers are unaware of their own cognitive limitations in driving situations that demand greater mental workload, such as overtaking. This presents an opportunity for education regarding maturational limitations and capabilities of young drivers and associated crash risk. Given that overtaking is an area also identified as a key driving practice associated contributing to increased crash risk around trucks, particularly due to limitations of trucks with regards to stopping distance [3,4], there may be benefit in incorporating this information into educational initiatives aimed at reducing crash risk of young drivers around trucks.

### 5.3. Young Driver Risky Driving Behaviours

Crash analysis research assessing collision culpability of young drivers in injury-causing multivehicle collisions suggests that young drivers are at fault in 92% of cases [62]. The literature suggests that four risky driving behaviours are associated with this increased crash risk for young drivers: the presence of peer passengers, speeding, night driving, and driver inattentiveness [62,63,64]. The risk associated with peer passengers is an important consideration with regards to increased crash risk around trucks, as the presence of peer passengers independently increases the crash risk for young drivers [65]. It is also associated with other risky driving behaviours, including speeding [66] and driver distraction [66]; factors that may compound the risk of collision with other vehicles, including trucks. Speeding, night driving, and driver inattentiveness are also foci as each of these factors is known to independently increase crash risk for young drivers [63]. It is reasonable to expect, therefore, that if young drivers are engaging in these risky driving behaviours around trucks, the risk of a collision between trucks and young driver vehicles may be compounded [13].

#### 5.3.1. Peer-Aged Passengers

For young drivers, the presence of a single peer-aged passenger can increase the risk of collision by 2.5 times and the presence of two or more peer-aged passengers increases the likely crash risk by 5.5 times [64]. Comparatively, older drivers (>25 years) appear to have no increased crash risk due to passenger presence, regardless of passenger age [65]. The presence of peer-aged passengers also increases the odds of car crash injury for young drivers, with the presence of one peer-aged passenger increasing the odds of driver injury 10.19 times, and the presence of two or more peer-aged passengers increasing the odds of driver injury 15.55 times [65].

Interestingly, young drivers have little awareness that the presence of peer-aged passengers increases their collision risk [67]. Given that risk of serious injury or fatality is greater for passenger vehicle drivers and occupants than for truck drivers [3], truck safety education initiatives could potentially include raising young driver awareness of increased collision risk with trucks due to the direct influence of peer-aged passengers, as well as how the presence of peer-aged passengers increases collision risk associated with speeding, night driving and driver inattentiveness for young drivers [64,65,66,67,68]. In addition, education initiatives could educate young drivers about the greater severity of consequences for both the driver and their passengers resulting from a collision with a truck versus a light passenger vehicle, such as greater likelihood of death or serious injury for passenger vehicle occupants than for occupants of trucks.

#### 5.3.2. Speeding

Driving at an unsafe speed is a critical hazardous driving act known to contribute to increased frequency of passenger vehicle-truck collisions, and greater risk of serious injury or death [4]. It is also a critical factor predicting collision for young drivers. Research investigating young driver collision culpability in multivehicle collisions suggests that speeding was a critical factor in 80% of cases, when young drivers were responsible for a collision [62].

Research into drivers’ intent to speed, based on the Theory of Planned Behaviour [69] indicates that young drivers are more likely to speed compared to older drivers [70]. Most prevalent factors associated with actual speeding prevalence among young U.S. drivers are “sensation seeking, substance use, tolerance of deviance, susceptibility to peer pressure, and … risky friends”, with no significant difference found between young male and female drivers [66] (p. 397). A study of French drivers suggests that key predictors of speeding in young drivers are sensation seeking and driver anger [71]. In Australia, the most significant predictors of speeding for young novice drivers are “gender, car ownership, reward sensitivity, depression, personal attitudes, and learner speeding” [72] (p. 242). Observable differences in the psychosocial factors predicting speeding behaviour for young drivers in Australia versus those in France and the U.S. suggests that educational initiatives aimed at targeting speeding behaviour of young drivers may need to be tailored for specific demographic populations to ensure that underlying factors are appropriately addressed.

Passenger-vehicle driver speeding is identified as one of the top four factors associated with fatal passenger-vehicle/truck collisions [5]. When we consider that young drivers have increased crash risk associated with speeding [62], as well as less likely developed situation awareness and hazard perception skills around trucks than more experienced drivers [51,52], a lack of experience is likely to result in young drivers having a reduced ability to react to dangerous driving situations around trucks when they are speeding, thus increasing crash risk around trucks. An absence of literature in this area does not preclude logical conflation of these factors, however further research is required to gain a comprehensive understanding of the extent to which young driver speeding behaviour may increase collision risk around trucks.

#### 5.3.3. Night Driving

Night driving is associated with increased crash risk for both truck drivers and young drivers, and there has been a considerable amount of research conducted in this area for both driving cohorts [23,73,74,75]. Analysis of 2019 Australian crash fatality data shows that 43% of collisions between trucks and young drivers (17–25 years) occurred at night, whereas only 28% of collisions between trucks and older drivers (over 25 years) occurred at night [76]. This indicates that young drivers are more likely to be involved in collisions with trucks at night time, compared to older drivers. However, there is little consideration in the literature of the specific risk of collision between trucks and young drivers at night, or how human factors may contribute to this risk. Here we focus on the increased risk associated with night driving for young drivers, from within the framework of risky driving behaviour, and consider how this might impact young driver behaviour around trucks at night.

Key factors contributing to increased risk of collision at night for young drivers appears to be related to sleepiness and driving under the influence of alcohol [73]. While there are licensing restrictions regarding alcohol use when driving in Australia, including a zero blood alcohol content limit for all provisional drivers, there are no provisional driving restrictions around driving at night in three of the eight Australian states and territories. With the exception of Western Australia, night driving restrictions in most jurisdictions relate only to the presence of passengers, but do not prevent provisional licencees from actually driving at night [40]. Night-time driving restrictions are imposed on provisional drivers in other countries, such as the Unites States, and evidence suggests that this does reduce young driver crash risk by up to 10% [73]. One way to avoid night-time crash risk for young Australian drivers may therefore be to implement night-time driving restrictions for provisional drivers. In the absence of such restrictions, educating young drivers about the impact of sleepiness on driving behaviour, with particular attention to increased risks associated with night driving, may be another avenue for reducing risk of collision for young drivers at night.

Some research suggests that young drivers appear to have little awareness that their crash risk increases when driving at night [67], while other research suggests that drivers aged 16–30 have a similar level of awareness of increased crash risk when driving at night, despite having different levels of driving experience [77]. Research regarding young driver behaviour at times of increased sleepiness shows that young drivers do have an awareness that increased subjective feelings of sleepiness are associated with increased crash risk, however, knowledge of this risk associated with increased sleepiness does not necessarily prevent young drivers from driving when sleepy [78]. In their study, Smith, et al. [78] required young drivers to maintain a four week sleep diary and a four week driving diary, as well as to note their subjective sleepiness for each driving episode and their perceived crash risk. Although drivers acknowledged that crash risk increased as subjective sleepiness increased, results showed that young drivers frequently chose to drive at times of predicted sleepiness and at times when they themselves felt sleepy.

Analysing young driver’s night driving through the lens of truck safety is important primarily because truck crash incidences are more likely to be severe at night compared with morning or day-time incidences [79]. A contributing factor here is that long-haul solo truck drivers in Australia are allowed to drive for up to 72 h a week, with the only restriction being that the driver must take a 24 h continuous stationary rest break during this period. There is no apparent restriction regarding the times of day that the driver can or cannot drive [80]. A full analysis of fatigue in long-haul truck drivers is beyond the scope of this paper, however clearly the separate risk factors of night-time driving with trucks on one hand, and young drivers on the other, compound to suggest that young driver collision risk with trucks increases night time, and risk of death or serious injury is greater at this time.

#### 5.3.4. Inattentiveness due to Smartphone Use While Driving

In Australia, hand-held mobile (cell) phone use while driving is illegal and can result in substantial penalties. Restricted licence holders–including learner and P1 and P2 provisional drivers–are not permitted to use their phones at all while driving, and this restriction extends to use of hands-free or Bluetooth functions. Current penalties result in a loss of licence for learner and P1 provisional drivers if smartphone use while driving is detected [81].

Despite the hefty penalties for using a smartphone while driving, and potential loss of licence, young drivers continue to use smartphones. Alarmingly, one study demonstrated that young drivers continue to use their smartphones to talk and text while driving, despite having an awareness that doing so increases their risk of collision [82]. Research suggests that a motivation for young drivers to use their smartphones is associated with a need to engage in social interactive technologies that facilitate communication (e.g., Snapchat, Twitter) [30]. A recent survey of 18–25 year old drivers sought to gauge daily use of social interactive technologies while driving, and found that 64.9% of respondents reported monitoring 1 to 5 social communications while driving, and 37.8% of respondents reported responding to 1 to 5 social communications while driving [30].

Of concern, it has been shown that increased functionality of smartphones may be encouraging drivers to use them in the more dangerous hand-held mode [83] thereby concealing their use from outside view and making detection and enforcement difficult [84]. This is of particular concern with regards to collision risk, as the driver’s eyes are diverted further from the road and down towards the smartphone. This visual diversion reduces the young driver’s ability to remain focused on scanning for potential hazards while driving. A driver simulation study has demonstrated that novice drivers aged 18–21 years spend 400% more time looking away from the road when texting than when they are not texting [85]. The consequences of not remaining focused when driving around trucks, as we have noted, can therefore be fatal for young passenger-vehicle drivers and occupants.

Driver distraction due to smartphone use has been found to increase the likelihood of a collision by up to four times [86,87], and increase likelihood of associated road trauma injury [88]. When we consider that: (i) drivers aged 18–25 years are more likely to engage in smartphone use while driving than any other age group [89], (ii) engaging in smartphone use is associated with increased driver inattentiveness [82], and (iii) passenger vehicle driver inattentiveness is one of the main factors identified by truck drivers as contributing to increased collision risk with a truck or heavy vehicle [5], it is reasonable to conclude that the prevalent use of smartphones by young drivers is likely to exacerbate crash risk between trucks and young drivers. This is an area of concern that requires further research to understand the extent to which young driver smartphone use increases risk of a collision when driving in the vicinity of trucks. Given that smartphone use while driving persists despite both hefty penalties [81] and young driver awareness of increased crash risk [82], further research is also needed to investigate how educational initiatives might increase young drivers’ knowledge of the increased collision risk around trucks due to driver inattentiveness associated with smartphone use while driving. Targeted educational initiatives should also incorporate content that facilitates behavioural change with regards to smartphone use while driving.

## 6. Current Educational Initiatives Targeting Safe Driving Practices around Trucks for Young Drivers

There is currently little available information for young drivers regarding road safety considerations that specifically relate to sharing the roads with trucks and other heavy vehicles. What is needed is practical information that increases young drivers’ situation awareness and hazard perception around trucks, as well as resilience-based educational initiatives that focus on reducing crash risk associated with risky driving behaviours of young drivers. Evidence from a prospective, longitudinal study of over 20,000 young drivers aged 17–24 in NSW, Australia, found that resilience-focused programs aimed at reducing risk-taking behaviours was associated with a 44% reduced relative crash risk [36]. This suggests that educational initiatives aimed at informing young drivers how to safely share the roads with trucks needs to not only provide factual information regarding awareness of the performance limitations of trucks, but also needs to consider how to target and curb potentially risky driving behaviours of young drivers around trucks that are known to increase crash risk. Here we provide a brief overview and critique of current young driver educational initiatives and make recommendations for future educational endeavours.

### 6.1. Graduated Driver Licensing Programs in Australia

Although not specifically a truck awareness initiative, graduated driver licensing programs are designed to ensure that young drivers are introduced to driving in an incremental manner, and situations that involve the most risk, such as driving at high speeds, driving at night, and driving with peer passengers, are not introduced until the young driver has gained fundamental driving skills in relatively lower risk road traffic conditions. A systematic review of graduated licensing schemes in the US, Canada, New Zealand and Australia reveals that graduated licensing schemes vary greatly across and within nations, and quality of the programs impacts overall effectiveness [29]. Yet, despite differences in quality of graduated licensing programs, all programs that were reviewed were found to reduce overall crash risk for young drivers [29]. Here we provide a brief overview of the graduated driver licensing initiatives in Australia, discuss how such initiatives contribute to truck awareness, and identify limitations of graduated driver licensing programs with regards to communicating knowledge around truck safety.

Australian graduated driver licensing programs are independently administered by each state and territory, and each provides different road rule information to young drivers that is aligned with specific state and territory road laws [40]. Although there is variation in the duration of graduated driver licensing stages, all graduated driver licensing programs in Australia typically include a pre-learner stage, followed by driver’s obtaining their permit where they are required to record a minimum number of log-book hours. After passing their Driver Licence Test, which is comprised of a written knowledge test and a practical driving test, newly licensed drivers can drive independently under a provisional driver licence that typically lasts 3 years.

A cursory review of road user handbooks in Australian states and territories reveals that very little attention is directed towards educating users about the limitations of trucks and the implications of such limitations for other road users. Furthermore, Australian graduated driver licensing programs provide very little standardised information or instruction regarding hazard perception and situation awareness around trucks. For example, the NSW Road User Handbook dedicates approximately 3–4 pages of the 212 page manual to truck limitations and truck safety, and while it provides an overview of key driver precautions around trucks, it does not provide great detail regarding truck limitations [18]. Dissemination of information regarding trucks and truck safety is largely left to individual driving instructors, and given that learner drivers can receive instruction from accredited driving instructors or via informal driving instruction from another licensed driver (usually a parent), this adds a great deal of variability to the way in which information is disseminated to young drivers regarding truck limitations and associated motorist considerations necessary to ensure safe driving practice around trucks.

### 6.2. Persuasive Messaging Targeting Risky Driving Behaviours of Young Drivers

Persuasive messaging in the road safety context attempts to influence drivers to stop engaging in risky driving behaviours (e.g., using their smartphone while driving) and employ safer road user behaviours [90]. Such messaging is often designed to include negative consequences associated with risky driving behaviours, to elicit fear in the audience [90]. Recent research, however, has suggested that many drivers are growing numb to traditional fear appeals and it may be wise to broaden the scope of the emotional appeal to include positive appeals (e.g., [91]). Indeed, young male drivers, in particular, may find messages that elicit positive affect (e.g., pride, humour) more effective than those that elicit negative emotion [30,92]. Psychosocial research reveals that targeted driver education and persuasive messaging that is derived from theoretical and empirical research is key to reducing collision risk [93,94,95,96]. Road safety public education messages, however, have been criticised due to various factors including a lack of guiding theory, lack of audience segmentation to target individual subpopulations (e.g., young drivers), and lack of a rigorous scientific evaluation [94].

The Step Approach to Message Design and Testing (SatMDT; [94]) is a framework to guide the development of road safety messages and addresses these aforementioned criticisms. It is based on social-psychological theories of decision making, attitude-behaviour relations, and persuasion (e.g., Theory of Planned Behaviour [69], Extended Parallel Process Model [97]) and includes other evidence-based factors that have been shown to improve message effectiveness (e.g., inclusion of strategies in the message to reduce the risky behaviour, an emotional response to the message, and modelling of behaviour). Among other target behaviours, the SatMDT has successfully guided the development and evaluation of effective messages targeting smartphone use among young drivers (e.g., [30]) and speeding among young male drivers [95]. Of note, both these studies found that males are more likely to be persuaded by messages that elicit positive emotion, such as pride [30,95] whereas females are more likely to be persuaded by messages that elicit negative emotion, such as anxiety [30]. The use of a theoretical and evidence-based framework such as the SatMDT would be beneficial to target the factors associated with increased crash risk among young drivers around trucks.

### 6.3. Industry-Focused Truck Awareness Initiatives

As we have discussed, current approaches that are focused on reducing crash risk for young drivers, such as graduated driver licensing programs and persuasive messaging initiatives around road safety have resulted in measurable reduction in young driver collisions. While graduated driver licensing programs are shown to reduce crash risk for young drivers, they give little consideration to increased crash risk specifically regarding driving around trucks or other heavy vehicles. This results in young drivers having a knowledge gap regarding the increased hazards that need to be considered when driving in the vicinity of trucks [13]. Industry stakeholders have attempted to address this gap in information by providing education to young drivers around the potential dangers associated with driving around trucks. This would ideally improve young driver situation awareness and hazard perception around trucks and lead to safer driving practice around trucks. Here we provide a brief overview of these industry initiatives.

The Australian Trucking Association (ATA), in collaboration with partner organisations, developed an educational initiative called SafeT360, which is a custom-built truck that is fitted with interactive education stations that can visit schools and other learning institutions to provide immersive and engaging education on the known performance limitations of trucks [98]. For example, the SafeT360 truck has interactive virtual reality stations that put users in the truck driver seat, showing a truck driver’s perspective of the road. From this perspective, users gain a first-hand experience of the visibility limitations of truck drivers, and become aware of where passenger vehicle drivers, pedestrians and cyclists might need to position themselves on the road to avoid being in known truck blind spots. The SafeT360 truck also provides education around truck stopping distances, delivers education around the dangers of passenger vehicle drivers engaging in some risky driving behaviours such as distracted driving, and provides vignettes that discuss the increased serious injury and death resulting from collisions involving trucks [98]. This initiative gives young drivers insight into the interactions between trucks and other road users, and due to it being a mobile resource, it can reach young drivers in urban and regional areas throughout Australia. While this initiative offers valuable road safety information to young drivers, the next step in this initiative is a thorough outcomes evaluation to establish how engagement with SafeT360 truck safety education leads to observable changes in young driver knowledge and driving behaviour, and to determine what the consequent reduction in collision risk is between trucks and young drivers.

The NSW National Roads and Motorists’ Association (NRMA) has produced some driver training resources aimed at improving driver awareness around trucks, including videos such as “Top 10 tips from a Truckie”, and provides links on their website to external resources on sharing the road with heavy commercial vehicles [99]. Similarly, industries, research institutions and creative media agencies have collaborated to promote safety information videos to promote awareness of trucks and truck safety, and provide information about sharing the roads with heavy vehicles. For example, collaborations with the Royal Automobile Club of Queensland resulted in production of a short video aimed at encouraging safe driving practices around trucks [100] and similar truck safety media was created under the Re:act collaborative project [101]. While these initiatives provide valuable road safety information around trucks, the dissemination of these materials is largely dependent upon users searching online to seek out such resources, and there is no evaluative measure of their effectiveness in terms of changing road user behaviour.

The overarching theme evident with these industry programs is that they provide valuable information to young drivers about trucks and heavy vehicles, which would likely improve young driver situation awareness and hazard perception, but there is no measurable evaluation of their effect. This highlights the need for robust and thorough evaluation of the demonstrable educational outcomes, as included in the SatMDT framework outlined above. For example, evaluation measures could focus on changes in young driver knowledge of the limitations of trucks and awareness of the necessary considerations of other motorists around trucks. In addition, these initiatives are likely to be more effective in reducing passenger vehicle-truck collision risk if they identify and target risky driving behaviours of young drivers that are known to increase collision risk with trucks.

## 7. Recommendations for Future Work

### 7.1. Future Education Initiatives

There is a clear need for a more targeted approach to young driver truck awareness, in addition to that which is provided via graduated licensing programs, to provide specific information about the limitations of trucks and build knowledge about safe driving practice around trucks. While there are some industry-led targeted educational initiatives that aim to reduce crash risk between trucks and young drivers by increasing young driver knowledge of the limitations of trucks, the effectiveness of these initiatives has not yet been evaluated. It may be that such initiatives will have greater effectiveness if they incorporate messaging that aims to target young driver risky driving behaviours around trucks, but this is yet to be determined. We suggest that for road safety education to be successful in changing young driver behaviour around trucks, education and instruction needs to be designed based on robust theories and empirical evidence so that the safety messages are effectively heard and received by the target audience and, ultimately, result in driver behaviour change. This requires a knowledge of the human factors that influence young drivers’ risky behaviour, such as those outlined in this review paper. Future initiatives must also be informed by empirical research regarding approaches that are likely to be effective in promoting safe driving behaviour for young drivers specifically. We suggest that real change in young driver intentions and behaviours can be best achieved through a collaborative approach between industry stakeholders and research institutions, to ensure that initiatives are informed by good science and theoretical frameworks, and are appropriately evaluated and modified for maximum educational effect.

### 7.2. Future Research Initiatives

We recommend that future research initiatives should be aimed at gaining an understanding of young drivers’ situation awareness and hazard detection around trucks and heavy vehicles, so as to inform educational initiatives that may be aimed at changing young driver behaviour to improve road safety around trucks. Dash-cam footage and portable eye-tracking technology can be used to measure young driver behaviour around trucks in natural driving situations, and provide real-time data. These data can be analysed in conjunction with survey data that measures what young drivers know about the limitations of trucks and the necessary safety precautions that should be taken by passenger vehicle drivers to avoid collisions around trucks. This would provide an opportunity to gain an empirical understanding of how young driver knowledge of trucks and truck safety might influence their own driving behaviour around trucks, and would inform potential road safety initiatives that aim to promote truck awareness. Additionally, young driver behaviour can be measured and monitored in simulated naturalistic (video-recorded) driving environments, using eye-tracking technology to explore dwell time, scanning behaviour and gaze patterns as measures of attentional allocation and situation awareness in simulated driving scenarios involving trucks, where young drivers can be trained to react to potentially hazardous situations involving trucks in a safe environment. Finally, we recommend that research be conducted to investigate young drivers’ intention to change their driving behaviour, if they are found to exhibit dangerous driving behaviours that may increase the potential likelihood of being involved in a collision with a truck or heavy vehicle.

## 8. Conclusions

There are a multitude of factors that influence a passenger-vehicle driver’s ability to safely share the road with trucks and heavy vehicles. This narrative review predominantly focused on bringing together research on the human factors and risky driving behaviours that influence young driver safety with research regarding hazards and limitations of trucks that are known to contribute to increased likelihood of collision, to then postulate which human factors might most likely increase young driver crash risk around trucks. Of particular interest is the potential interactive relationship between the known limitations of trucks and the known limitations of young drivers. Specifically, this review focused on investigating the perceptual limitations of young drivers, which are likely to restrict a young driver’s ability to detect hazards specific to trucks, as well as the known risky driving behaviours of young drivers that that can potentially compound their risk of collision with trucks. Research and education initiatives were recommended to investigate these potential interactive collision risks and provide practical solutions that might minimize collision risk between young drivers and trucks, and associated risk of serious injury or death, particularly for passenger vehicle occupants.

## Data Availability

Not applicable.
